# Clinical and mechanical factors associated with the removal of temporary epicardial pacemaker wires after cardiac surgery

**DOI:** 10.1186/s13019-016-0414-2

**Published:** 2016-01-16

**Authors:** Elsayed Elmistekawy, Yen-Yen Gee, Dai Une, Matthieu Lemay, Anne Stolarik, Fraser D. Rubens

**Affiliations:** University of Ottawa Heart Institute, 40 Ruskin St., Ottawa, Ontario K1Y 4 W7 Canada

**Keywords:** Epicardial pacemaker wires, temporary pacing, complications, cardiac surgery

## Abstract

**Background:**

Temporary pacemaker wires are placed in the majority of patients after cardiac surgery. There is no information on mechanical factors related to wire removal.

**Methods:**

Clinical information related to temporary wire use and removal was prospectively collected from a large cardiac surgical unit over one year. Measurements of maximal tension that nurses and doctors would apply to remove temporary wires was determined using a hand-held portable scale. In a prospective trial, patients (*n* = 41) had their wires extracted in series to the portable scale to determine the maximal tension required for safe removal.

**Results:**

Ventricular wires were placed in 86.5 % of patients during the observed year. Pacing facilitated weaning from CPB in over 15 % of patients and pacer dependence was seen in 2.1 %. No patients suffered major complications after wire removal. There was no difference in the tension that physicians or nurses would apply to comfortably extract temporary wires. In the prospective trial, there was no difference in the tension required for removal of atrial or ventricular wires (atrial 18.3 ± 17.9 oz versus 14.5 ± 14.2 oz, *p* = 0.430). There were no patient factors that correlated with the degree of resistance and there was no significant difference between the tension required to remove wires with (21.0 ± 22.5 oz) or without (14.1 ± 5.1 oz) an atrial button.

**Conclusions:**

Temporary epicardial wire removal is innocuous and was not associated with any complications. In some patients tension required for safe removal exceeded 20 ounces. Strategies to standardize wire removal may prevent complications and may minimize unnecessary wire retention.

## Introduction

Temporary epicardial pacemaker wires are routinely inserted in the vast majority of patients after cardiac surgery. Though infrequently used, they can be an essential adjunct to facilitate weaning from cardiopulmonary bypass and they may be life saving in the situation of early or late postoperative heart block [1].

Wires are generally removed close to the time of hospital discharge, often as a designated medical act by a nurse. In the majority of cases, this procedure is well-tolerated by the patient however infrequently complications may occur such as late tamponade. Further, failure to remove a temporary wire completely has been associated with late complications such as infection [2] and wire migration [3].

There is no clinical information to guide medical practitioners how to optimally remove temporary wires. We sought to assess the tension required for the routine removal of temporary epicardial pacemaker wires after cardiac surgery as well as factors related to resistance of removal. We also sought to evaluate factors related to routine use including their need during weaning from cardiopulmonary bypass (CPB) and complications related to their removal.

## Methods

### Ethics

Approval for this project was obtained from the Ottawa Hospital Research Ethics Board. Individual informed consent was obtained from patients participating in the study. With regards to the aggregate data of patients, the University of Ottawa Heart Institute has approval from its institutional research ethics board to anonymously publish data that are prospectively collected before and after cardiac surgery. As such, individual patient consent for this portion was waived.

### Study Patients

#### Aggregate Data

We retrospectively analyzed prospectively collected data from the University of Ottawa Peri-Operative Database Unit to identify patients undergoing cardiac surgery between 02/01/2014 and 31/12/2014 (*n* = 1582). The database captures detailed information on preoperative, peri-procedural and postoperative variables for all patients undergoing cardiac surgery and it is maintained by a team of full-time data abstractors who are responsible for data collection and an ongoing audit process.

#### Determination of Maximal Comfortable Tension

Volunteers from the attending surgical staff (senior housestaff and staff surgeons) as well as nursing staff experienced in temporary wire removal were asked to apply tension to a loop of wire attached to the hand-held portable electronic scale to document the maximal weight tension at which they would cease further pulling.

#### Removal Tension Determination

Patients undergoing urgent or elective cardiac surgery were eligible for participation. Contraindications to inclusion included emergency surgery and non-sternotomy incisions. Temporary epicardial pacemaker wire (Streamline™, Medtronic, Minneapolis, MN) insertion was left to surgeon preference for both atrial and ventricular leads. Polyethylene buttons were used for some atrial wires and other wires were attached to right atrium with hemoclips.

Pacer wires were removed at discharge or at a maximum of 4–6 weeks if the patient was still in hospital. Removal of wires of patients on Coumadin was only completed when the INR was less than 2.5. At the time of wire removal, a loop was fashioned with the exterior end of the wire. The wire was then removed in series with an interposed hand-held portable electronic scale (Chestnut Tools, Almonte, Ontario) to measure the maximal tension required. After removal, patients were observed for 4 hours.

#### Statistical analysis

Continuous variables were reported as mean ± SD or median ± interquartile range for non-normally distributed continuous variables. Categorical variables were reported as counts and percentages. Student’s unpaired t tests with unequal variances were used to compare continuous variables between groups. Linear regression was used to assess factors associated with the tension of atrial and ventricular wire removal. Logistic regression was used to assess factors associated with tensions greater than 20 ounces to remove ventricular wires. Factors tested included age, time from surgery to removal (days), age, weight, procedure and body surface area. Values with p values <0.1 were tested in multivariable models if appropriate. A *p* value <0.05 was considered significant. All statistical analyses and plots were performed with Stata® version 13.1 (College Station, Texas).

## Results

### Aggregate Data

The characteristics of the 1582 patients are presented in Table 1. Ventricular wires were placed in 1368 patients (86.5 %) whereas atrial wires were used in 580 patients (36.7 %). Atrial and ventricular wires and sequential wires were used in weaning from CPB in 47 (3.0 %), 61(3.9 %) and 131(8.3 %) respectively (total 15.2 %). Pacer dependence during CPB weaning was demonstrated in 33 patients (2.1 %). These cases included 27 % post CABG/AVR, 24 % post CABG, 12 % post transplant, 12 % post AVR Maze procedure and 24 % other.

**Table 1:** One year aggregate data

Patient Characteristics (n = 1582)	
Age (yrs)	67.6 ± 12.5
Male gender n(%)	1108 (70)
Urgency n(%)	
Elective	858(54)
Urgent	595(38)
Emergency	129(8)
Reoperation n(%)	134(8)
Procedures (%)^a^	
CABG	59.6
Aortic Valve Replacement	29.5
Mitral Repair	9.5
Maze	7.7
Off-Pump CABG	6.9
Combined	30.1
Other^b^	`44.3

^a^Summation >100 % due to combined procedures

^b^Includes mitral replacement, trans-aortic valve replacement, tricuspid surgery, aortic surgery, aortic root replacement, transplant, pulmonary thromboendarterectomy, assist devices

Abbreviations: CABG-coronary artery bypass grafting

Pacer wires were removed by a physician in 2.9 % of patients and by nurses in 97.1 %. Wires were clipped in 104 cases (6.6 %) due to excessive resistance (56 [53.9 %]) or due to caution related to documented suturing of the wire to the epicardium (48 [46.2 %]). Problems documented in the nursing notes included resistance (57 [3.6 %]), minor bleeding (19 [1.2 %]) and patient discomfort (12 [0.8 %])

### Determination of Maximal Comfortable Tension

**Fig. 1:**
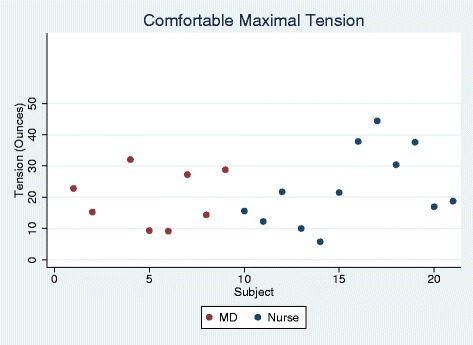
Comfortable maximal tension (ounces) – Medical staff were asked to pull on a wire attached to the tensiometer. Measurements in triplicate with average presented.

There maximum tension applied by experienced nurses (*n* = 12) as compared to physicians (*n* = 8) was not significantly different (nurse 22.7 ± 12.2 ounces, MD 19.9 ± 9.0 oz, *p* = 0.579). The distribution of the tension applied is illustrated in Fig. 1.

### Removal Tension Determination

**Fig. 2:**
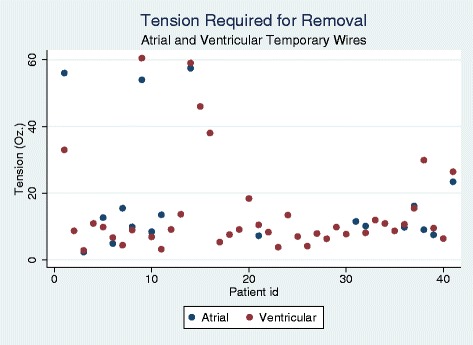
Tension (ounces) required for removal ventricular and atrial wires from patients at discharge.

Patient characteristics are presented in Table 2. 97.6 % of patients were on acetylsalicylic acid (ASA) at the time of removal. Sixteen patients (39.0 %) were on Coumadin at the time of removal with an average INR of 2.1(1.7, 2.6). Atrial buttons were used in 11 patients with atrial wires (57.9 %). There was no difference in the tension required for removal of atrial or ventricular wires (atrial 18.3 ± 17.9 oz versus 14.5 ± 14.2 oz, *p* = 0.430). The tension required for wire removal is presented in Fig. 2. No factors were identified by linear regression to be associated with the tension required for either atrial or ventricular wire removal (results not shown). Multivariables tested for factors predictive of required tension >20 ounces with *p* < 0.1 in the univariable models included weight (OR 0.948 95%CI [0.887-1.013, *p* = 0.114) and time from surgery (OR 1.085 95%CI [0.984-1.195] *p* = 0.101). There was no statistically significant difference between the tension required to remove wires with (21.0 ± 22.5 oz) or without (14.1 ± 5.1 oz) a button.

**Table 2:** Tension removal trial

Patient Characteristics (*n* = 41)	
Age (yrs.)	65.5 ± 11.9
Male gender n(%)	38(92.7 %)
Height (cm)	174.9 ± 10.2
Weight(kg)	86.1 ± 14.8
BSA (kg/m^2^)	2.01 ± 0.19
Wire removal (days [IQ])	6 [5, 9]
Procedures n(%)	
CABG/OPCAB	21(51.2)
MV repair	5(12.2)
CABG AVR	4(9.8)
AVR	3(7.3)
MV replacement	3(7.3)
Aortic root surgery	1(2.4)
AVR MV replacement	1(2.4)
CABG aortic root surgery	1(2.4)
CABG AVR MV repair	1(2.4)
Aortic root MV repair	1(2.4)

Abbreviations: IQ – interquartile, CABG – coronary artery bypass grafting, OPCAB-offpump coronary artery bypass, MV – mitral valve, AVR – aortic valve replacement

## Discussion

Temporary epicardial pacemakers were infrequently used for weaning from CPB but were essential due to heart block in 2.1 % of patients. Removal of epicardial wires at the time of discharge was uncomplicated in the majority of patients. There was no significant difference in the amount of tension that nurses or physicians deemed was comfortable for temporary wire removal. In the prospective patient study, there was no difference in the tension that was required between atrial and ventricular wire removal. The majority of the wires were removed with less than 20 ounces of tension. No patient factors were statistically associated with the need to apply greater than 20 ounces of tension.

Ventricular temporary epicardial wires are generally inserted through a superficial bite of the anterior wall of the right ventricle. There are multiple means by which wires may be affixed to the right atrium including direct suture or clipping, or through the use of an atrial button. Previous investigators have demonstrated that numerous patient factors may be associated with the subsequent need for temporary pacing such as advanced age, NYHA III-IV, pulmonary hypertension, digoxin use, multiple valvular replacement, long cross-clamp time and annular calcification [4]. In the majority of cases, pacer wires are not used and in fact, some surgical groups have recommended more selective insertion, particularly with low-risk surgery such as elective off-pump coronary bypass grafting [5].

Pacers wires were infrequently utilized for weaning from CPB in this study, but when used, a sequential pacing mode was used more often. Pacemaker dependence on weaning, seen in 2.1 % of all patients in a single year at our busy surgical unit, may be associated with a the subsequent need for insertion of a permanent pacemaker. This fraction is similar to that described by other authors who have also advocated more selective use [6].

Complications related to temporary pacemaker wire removal are uncommon. The most concerning issue relates to hemorrhage and late tamponade which may be life-threatening [7, 8].

There is no documentation of the safe maximal degree of tension that may be applied on a temporary atrial or ventricular wire on its removal after cardiac surgery. This tension has only been described in a subjective manner thus complicating teaching safe practice in this designated medical act. Pacer wires were clipped at the skin in 6.6 % of our patients. Problems related to wire retention include late patient discomfort, wire migration [3], organ herniation [9]and late infection [2]. Further, patients may be unable to undergo some forms of magnetic resonance imaging with higher magnetic fields (3 Tesla) due to concerns of heat transmission and cardiac damage [10]. Therefore measures should be taken to ensure that unnecessary wire retention is avoided.

The mean tension that health professionals identified as a comfortable maximum exceeded the mean tension for both atrial and ventricular wires, however, over 20 % exceeded this amount and thus these wires may have been cut and retained depending on the experience of the nurse of physician. No problems came of removal of these wires. Further there were no issues after removal of wires in the presence of a moderately elevated INR, even in the presence of ASA usage.

Limitations of the prospective patient experiment included single chamber wires in some patients such that duplicate measurements were not available. Not all patients had both atrial and ventricular wires however ventricular wire samples were available in all and it is our impression that removal from this site is most commonly associated with complications. More late complications may have occurred in the larger population such as sub-clinical pericardial effusions and these were not determined as patients did not undergo routine echocardiographic followup.

In summary, temporary epicardial pacemaker wires removal is generally innocuous. Tension required for removal is generally under 20 ounces and in there were no patient or procedure factors predictive of the degree of tension. Standardizing the tension required to remove wires may facilitate teaching of this procedure and may prevent early and late complications as well as possibly unnecessary retention of intracorporeal temporary wires after surgery if they are left due to perceived excessive resistance.

## References

[1] Reade MC (2007). Temporary epicardial pacing after cardiac surgery: a practical review: part 1: general considerations in the management of epicardial pacing. Anaesthesia.

[2] Dyal HK, Sehgal R. The catastrophic journey of a retained temporary epicardial pacemaker wire leading to Enterococcus faecalis endocarditis and subsequent stroke. BMJ Case Rep. 2015;2015. doi:10.1136/bcr-2014-206215. 10.1136/bcr-2014-206215 PMC4289793 25568268

[3] Mukaihara K, Yotsumoto G, Matsumoto K, Imoto Y (2015). Migration of a retained temporary epicardial pacing wire into an abdominal aortic aneurysm. Eur J Cardiothorac Surg.

[4] Alwaqfi NR, Ibrahim KS, Khader YS, Baker AA (2014). Predictors of temporary epicardial pacing wires use after valve surgery. J Cardiothorac Surg.

[5] Puskas JD, Sharoni E, Williams WH, Petersen R, Duke P, Guyton RA (2003). Is routine use of temporary epicardial pacing wires necessary after either OPCAB or conventional CABG/CPB?. Heart Surg Forum.

[6] Bethea BT, Salazar JD, Grega MA, Doty JR, Fitton TP, Alejo DE (2005). Determining the utility of temporary pacing wires after coronary artery bypass surgery. Ann Thorac Surg.

[7] Baldwin BJ, Dorney ER (1971). Acute cardiac tamponade following the removal of temporary epicardial pacemaker wires after open heart surgery. Am J Med Sci.

[8] Hoidal CR (1986). Pericardial tamponade after removal of an epicardial pacemaker wire. Crit Care Med.

[9] Shaikhrezai K, Khorsandi M, Patronis M, Prasad S (2012). Is it safe to cut pacing wires flush with the skin instead of removing them?. Interact Cardiovasc Thorac Surg.

[10] Pfeil A, Drobnik S, Rzanny R, Aboud A, Bottcher J, Schmidt P (2012). Compatibility of temporary pacemaker myocardial pacing leads with magnetic resonance imaging: an ex vivo tissue study. Int J Cardiovasc Imaging.

